# Aortic reimplantation of the superior mesenteric artery (SMA) for SMA stenosis in a previously stented patient: New technique and a case report

**DOI:** 10.1016/j.ijscr.2023.109170

**Published:** 2023-12-24

**Authors:** Mohammad Reza Zafarghandi, Alireza Samimiat, Parham Nikraftar, Afrooz Sadeghi, Pejman Pourazari, Amir Shokri

**Affiliations:** a Vascular Surgery, Department of Vascular Surgery, Sina Hospital, Tehran University of Medical Science, Tehran, Iran; b Department of General Surgery, Sina Hospital, Tehran University of Medical Sciences, Tehran, Iran; c Department of Vascular Surgery, Sina Hospital, Tehran University of Medical Science, Tehran, Iran; d Surgery Department of Surgery, School of Medicine Isfahan University of Medical Sciences, Isfahan, Iran; e Vascular & Trauma Surgery, Department of General Surgery, School of Medicine, Tehran University of Medical Sciences, Tehran, Iran

**Keywords:** Aortic reimplantation, Superior mesenteric artery stenosis, Previously stented patient

## Abstract

**Introduction and importance:**

Mesenteric artery stenosis leads to inadequate blood flow toward various parts of the gastrointestinal tract. Revascularization is the primary aim of treatment regardless of its approach. During the last decades, open revascularization has been replaced by endovascular-first approach. Mesenteric artery in-stent restenosis occurs in a considerable number of patients that need reintervention in up to half of them using redo endovascular revascularization or open surgery. Here, we reported a case of SMA and celiac artery stenoses treated by aortic reimplantation of the SMA.

**Case presentation:**

A 62-year-old man with history of previous stenting of CA and SMA was referred due to chronic intermittent abdominal. CT angiography of the abdomen showed restenosis of both arteries. A transection distal part of the occlusions SMA and reimplantation of it into the SMA on the anterolateral face of the infrarenal aorta as the end-to-side anastomosis were performed resulting in resolving the patient problem.

**Clinical discussion:**

Chronic mesenteric ischemia can result from various medical conditions. Mesenteric vascular surgical revascularization through open laparotomy had been considered the standard of care. However, minimally invasive surgery such as endovascular therapy has attracted attention in the recent decades. There are some concerns about the difficulties of further surgery in case of re-occlusion. The end-to-side anastomosis and aortic reimplantation can be considered in patients with appropriate runoff in the remaining parts of corresponding vessels.

**Conclusion:**

Aortic reimplantation of the superior mesenteric artery in patients with restenosis of stents is a viable option especially in case of inappropriate iliac artery to perform retrograde mesenteric bypass.

## Highlights

• Mesenteric artery stenosis leads to inadequate blood flow toward various parts of gastrointestinal tract
• Open revascularization has been replaced by endovascular-first approach
• Mesenteric artery in-stent restenosis occurs in a considerable number of patients
• Aortic reimplantation of the superior mesenteric artery in patient with restenosis of stents is a viable option.

## 1 Introduction

Mesenteric artery stenosis leads to inadequate blood flow toward various parts of gastrointestinal tract resulting in chronic mesenteric ischemia which mainly affects the small intestine [1]. More specifically, superior mesenteric artery (SMA) and celiac trunk stenoses originate from generalized atherosclerosis [2]. Their prevalence has been reported up to 11.2 % and 24 % in asymptomatic and symptomatic cases [[3], [4], [5]]. Nonetheless, combined celiac artery and SMA stenosis can be found in 7 % of cases [6]. While collateral vessels such as the pancreaticoduodenal arcades and the dorsal pancreatic artery help to maintain the blood flow of organs, the combined forms inhibit the efficacy of these collaterals [7].

Revascularization is the primary aim of treatment regardless of its approach. During the last decades, open revascularization has been replaced by endovascular-first approach, especially in patients at high surgical risk [8]. Despite the excellent outcomes of these minimally invasive approaches, such as angioplasty with or without stenting, mesenteric artery in-stent restenosis occurs in a considerable number of patients that needs reintervention in up to half of them using redo endovascular revascularization or open surgery [9].

Here, we reported a case of SMA and celiac artery stenoses suffering from symptomatic in-stent total occlusion treated by aortic reimplantation of the SMA. This study has been reported in line with the SCARE criteria [17].

## 2 Case

**Fig. 1:**
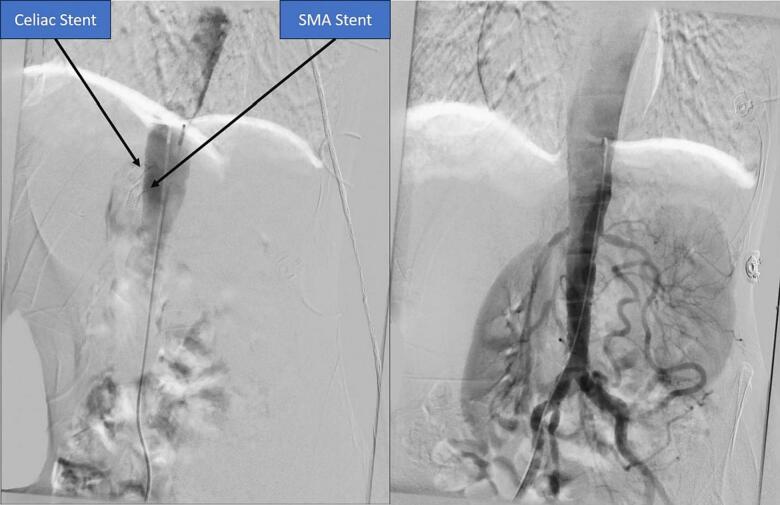
Abdominal angiography of patient before the surgery showing absence of blood flow in the stented SMA and celiac trunk.

**Fig. 2:**
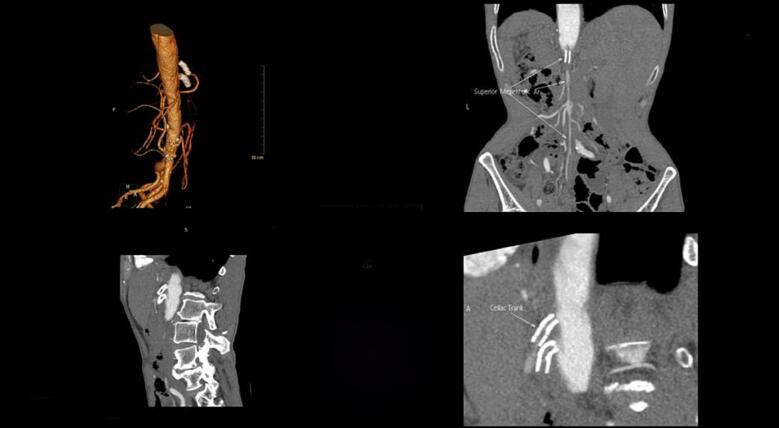
Abdominal CT angiography of patient before the surgery showing absence of blood flow in the stented SMA and celiac trunk and also run off of SMA.

**Fig. 3:**
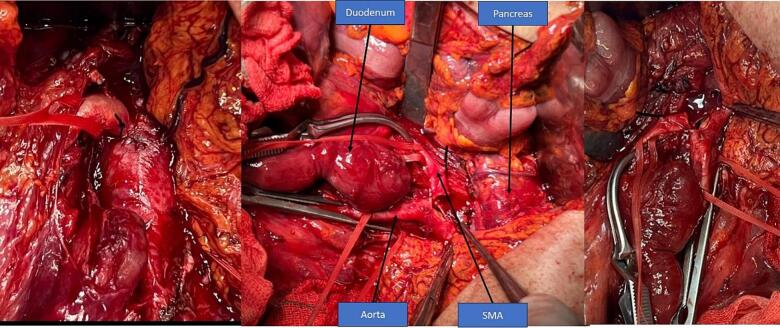
The surgical exploration of patient showing the obstructed SMA and celiac trunk.

**Fig. 4:**
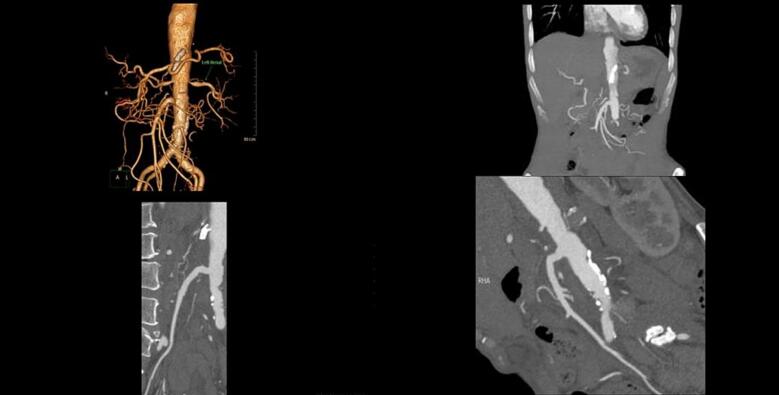
Abdominal CT angiography of patient one month after the surgery showing normal blood flow of the end-to-side anastomosis.

A 62-year-old man with history of chronic intermittent abdominal pain was presented to our department. The pain was postprandial and resolved by fasting. Moreover, he suffered from an unintentional weight loss, i.e., more than 50 % during the last two years. He underwent full investigation including hematology and blood chemistry tests, esophagogastroduodenoscopy, and colonoscopy revealing no specific diagnosis. However, concomitant stenoses of superior mesenteric and celiac arteries were detected at the ultrasound Doppler sonography and then computed tomography (CT) angiography of abdomen which reported 70 stenosis of SMA and celiac artery. Before the admission to our department, celiac and superior mesenteric artery stenting had been performed resulting in short-term relieving of symptoms. However, after six months, he complained of severe postprandial abdominal pain resulting in food intolerance. Abdominal CT angiography was repeated showing extensive and complete thromboses of both arteries and stents (Fig. 1, Fig. 2). Moreover, CT angiography showed appropriate runoff vessels and enough length of SMA in the remaining parts (two to three centimeters after the occulted stent). Endovascular approach was proposed; however, the patient did not accept the risk of restenosis and higher risks of open surgery in case of its occurrence. Subsequently, the patients underwent open laparotomy in the supine position with transmesenteric approach to perform retrograde C loop graft to the distal mesenteric artery. However, during the surgical exploration, an aneurysmal-like iliac artery was detected which was not an appropriate candidate for anastomosis and bypass the stenosis using dacron graft (Fig. 3). Therefore, it was decided to transection distal part of the occlusions SMA and reimplant the SMA on the anterolateral face of the infrarenal aorta as the end-to-side anastomosis. The patient was followed postoperatively every month by physical examination, showing a weight gain and after one- and six-month abdominal CT angiography was done which normal blood flow of the end-to-side anastomosis was reported (Fig. 4).

## 3 Discussion

Chronic mesenteric ischemia can result from various medical conditions including atrial fibrillation causing mesenteric artery embolism and atherosclerosis inducing mesenteric artery thrombosis. However, extensive collateral circulation helps the body to maintain blood flow to the affected organs mostly resulting in an asymptomatic disease even in the presence of several occluded vessels [3,10,11,and]. Eventually, a portion of patients will suffer from symptoms that are mainly related to meals resulting in postprandial intestinal angina. In these cases, the SMA is almost always occulted [12]. Mesenteric vascular surgical revascularization through open laparotomy has been considered the standard of care for the management of patients suffering from symptomatic mesenteric ischemia for a long time accompanied with a significant mortality rate. There are different open bypass techniques, which divided into two main groups, Retrograde mesenteric bypass and Antegrade mesenteric bypass. Recently an hybrid techniques using retrograde open mesenteric stenting via midline laparotomy to expose SMA has been reported by Milner and colleagues [[13], [14], [15], [16]]. However, minimally invasive surgery such as endovascular therapy which is performed percutaneous has attracted attentions in the recent decades [8]. Although, in-stent restenosis, which needs reintervention to mitigate the symptoms, remains as a major concern [9]. Despite the positive results of the application of minimally invasive surgery in the reinterventions for stent restenosis in patients treated for atherosclerotic mesenteric artery disease [9], there are some concerns about the difficulties of further surgery in case of re-occlusion. Therefore, open surgery, i.e., retrograde mesenteric bypass, is suggested by the most surgeons, routinely. However, in case of an inappropriate iliac artery to perform bypass, there are limited options to cure the patient. The end-to-side anastomosis and aortic reimplantation can be considered in patients with appropriate runoff in the remaining parts of corresponding vessels which is generally resulted from sufficient collateral circulation.

## 4 Conclusion

Aortic reimplantation of the superior mesenteric artery in patients with restenosis of stents is a viable option especially in case of inappropriate iliac artery to perform retrograde mesenteric bypass.

## Consent

Written informed consent was obtained from the patient to publish this case report and accompanying images. On request, a copy of the written consent is available for review by the Editor-in-Chief of this journal.

## Provenance and peer review

Not commissioned, externally peer-reviewed.

## Ethical approval

Ethical approval for this study (IR.TUMS.SINAHOSPITAL.REC.1402.088) was provided by the Ethics Committee of Tehran University of Medical Sciences, Tehran, IRAN.

## Funding

This research did not receive any specific grant from funding agencies in the public, commercial, or not-for-profit sectors.

## Author contribution

AFS and PP gathered and interpreted the patient data regarding history and physical examination. AS wrote the manuscript. MZ and ASH supervised and designed the project. MZ, PN and ASH perform surgery on the patient. AS, AFS and PP contributed to the discussion of the research. All authors have read and approved the manuscript.

## Informed consent

Written informed consent was obtained from the patient guardian for publication of this case report and accompanying images. A copy of the written consent is available for review by the Editor-in-Chief of this journal on request.

## Declaration of competing interest

The authors declare that they have no competing interests. Acknowledgments Not applicable.
